# Laparoscopic Radical Cystectomy and Ileal Neobladder for Muscle Invasive Bladder Cancer in Combination with One Stage Prophylactic Laparoscopic Sacrospinal Fixation to Avoid Future Pelvic Organ Prolapse

**DOI:** 10.1089/cren.2016.0029

**Published:** 2016-03-01

**Authors:** Péter Törzsök, Sophina Bauer, Rosemarie Forstner, Karl-Dietrich Sievert, Günter Janetschek, Reinhold Zimmermann

**Affiliations:** ^1^ Department of Urology and Andrology, University Hospital of Salzburg, Salzburg, Austria.; ^2^ Department of Radiology, University Hospital of Salzburg, Salzburg, Austria.

## Abstract

***Background:*** Women who undergo cystectomy with orthotopic ileal neobladder are more likely to have urinary retention and neocystocele mainly because of anatomical reasons than stress urinary incontinence. The risk is even higher in case of neurologic comorbidities, as in case of our patient.

***Case Presentation:*** We present a laparoscopic mesh insertion for sacrospinal colposuspension to prevent a neocystocele and pelvic organ prolapse in combination with laparoscopic radical cystectomy in a female patient suffering from bladder cancer and chronic episodic multiple sclerosis. After a 30-month follow-up, the patient is continent and voids without residual urine. A dynamic MR of the pelvis shows a minimal rectocele without any evidence of a cystocele.

***Conclusion:*** Laparoscopic cystectomy combined with sacrospinal mesh fixation is technically feasible and could be an option to prevent neocystocele for female patients.

## Introduction and Background

According to the European Association of Urology (EAU) guidelines,^1^ radical cystectomy (rCx) with orthotopic neobladder is one of the continent therapy options, with a good quality of life for women suffering from localized muscle invasive bladder cancer (miBC) without compromising the oncologic outcome.

The risk of neocystocele formation and, thus, the risk of neobladder dysfunction are commonly related to the prolapse of the vaginal stump because the suspension of the genital system is destroyed. Women who undergo rCx with orthotopic neobladder are more likely to have urinary retention.^2^ The mechanism of the urinary retention is still not completely clear. Such anatomical (pelvic organ prolapse [POP] with kinking of the urethra or neocystocele formation) and neural factors are suspected.^3^

Encephalomyelitis disseminata (multiple sclerosis [MS]) harbors the huge risk of bladder and pelvic floor dysfunction from the beginning. Innervation or, in general, neural problems could be even more predominant in those patients. Subsequently, they have hypothetically a higher risk to develop POP and neocystocele in the long run.

Sacrospinal mesh plastic is a therapy spread worldwide for POP.^4^ The sacrospinal ligament is one of the most sufficient structures for POP mesh anchoring. However, meshes for POP repair are debatable because of their inherent side effects and risk factors. The fixation technique and the size of the mesh are both crucial. The risk of erosion is theoretically even higher in patients with a neobladder. Bilateral-sacrospinous-colposuspension mesh (BSC mesh; weight 21 g/m^2^, porosity 93%) is a new mesh with a smaller and smoother surface and, thus, a lower risk for erosion, primarily made for open/vaginal POP repair. It includes a specific applicator to anchor the distal mesh arms properly within the sacrospinal ligament; to minimize and optimize the surgical approach because of the mesh properties, we considered it for laparoscopic pelvic floor stabilization at the time of laparoscopic rCx.

We present a female patient suffering from almost symptomless acute episodic MS and invasive bladder cancer (BC). She underwent laparoscopic rCx and orthotopic ileal neobladder. In addition, we combined the rCx with a simultaneous laparoscopic insertion of a colposuspension mesh to avoid pelvic organ and neobladder prolapse.

## Presentation of Case

We report on a 59-year-old female patient with miBC (pT2 N2 [2/26] R0 G3, low differentiated urothelial tumor) and comorbidities such as chronic episodic MS, collapse query cause, and chronic nicotine consumption. MS was fairly symptomless at the time of operation. She underwent a laparoscopic rCx in November 2012 with open orthotopic ileal neobladder. In addition, we performed an intraoperative laparoscopic sacrospinal fixation using a BSC mesh to avoid future POP.

The intraoperative course was generally without severe complications. Preoperatively the patient had no voiding/continence problems. Pad and residual urine tests were each negative. Vaginal examination excluded POP.

**FIG. 1.**
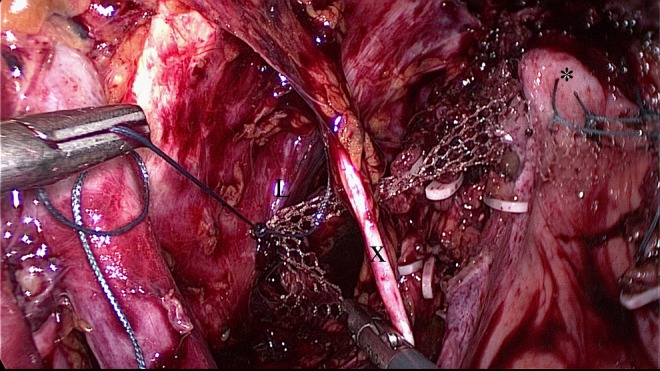
Placement of the BSC mesh onto the left sacrospinal ligament (L). The mesh is already fixed onto the vaginal vault (*). The mesh is placed under the obturator nerve (X). BSC mesh, bilateral-sacrospinous-colposuspension mesh.

The mesh we used for this very specific indication is relatively small and soft U shaped. It has a very low weight of 21 g/m^2^ and a high porosity (93%) to accelerate functional integration and minimize tissue reaction. It is actually made for the transvaginal approach for fixation onto the sacrospinal ligaments. The mesh was placed after cystectomy, the fixation was feasible by means of laparoscopy. The anterior part of the mesh was sutured onto the vaginal vault. The mesh arms could be properly fixed on the sacrospinal ligaments (Fig. 1). The ligaments could be defined clearly by laparoscopy coming from our experience with laparoscopic sacrocolpopexy. Although the mesh was not primarily designed for laparoscopy, it was easy to handle because of its characteristics. Additional OR time for mesh placement was <25 minutes. The placement was finally perfect to support the normal anatomy. The neobladder was subsequently formed by an open access and placed again by laparoscopy without any interference from the mesh. The postoperative course was uneventful and, in particular, without mesh complications so far.

## Follow-Up Results

After 36 months of follow-up, the patient is satisfied with her continence, she uses daily 0 to 1 small pad. During nights, the patient is mostly dry. She voids spontaneously and ultrasonograph showed no residual urine. The uroflowmetry showed a Q-max of 27 mL/second at a volume of 190 mL and a voiding time of 14.5 seconds. The most recent cystoscopy and the vaginal examination showed no abnormalities and, in particular, no POP.

**FIG. 2.**
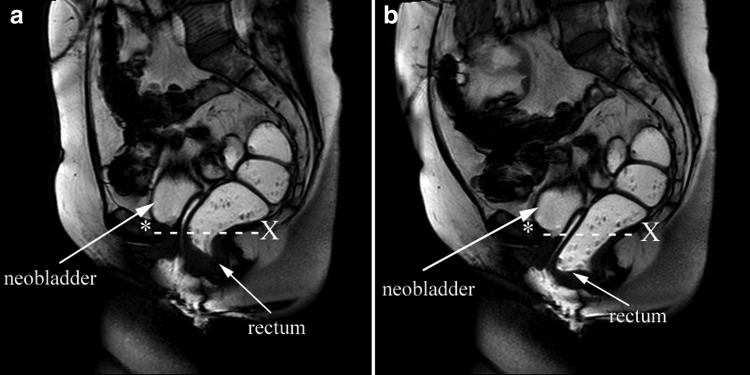
Dynamic MR of the pelvis. **(a)** At normal state. **(b)** At Valsava-attempt. *, os pubis; X, os coccygeum.

A dynamic MR of the pelvis showed no cystocele and a minimal rectocele grade I°. At Valsava-attempt reached the neobladder the pubococcygeal line (Fig. 2).

The patient has received four series of Gemcitabine/Carboplatine chemotherapy from June to September 2012 because of initial retroperitoneal lymph node metastases. The last staging CT from December 2015 showed small retroperitoneal lymph nodes paraaortic and no distant metastasis, that is, all in all stable disease.

## Discussion

According to the EAU Guidelines 2015, the choice of neobladder urinary division after rCx alone does not have significant influence on the oncologic outcome and results in a good quality of life in selected patients.^1^

Female patients after cystectomy (Cx) suffer urinary retention 25% and 50% after 5 years of follow-up. According to Ali-el-Dein and colleagues, the urinary retention was because of anatomical rather than functional reasons.^2^ All in all, potential pelvic floor impairment after Cx is a main factor for neobladder dysfunction, which is still a major limitation for this approach. POP in the female after Cx could even aggravate the preexisting problems with orthotopic neobladder. There are very limited data on pelvic floor dysfunction and POP in females with MS and no publication according to our best knowledge in the context of BC and rCx.

In case of POP, sacrospinal fixation shows excellent postoperative outcome with satisfaction of the patients. Thus, the “prophylactic” mesh placement could be a new option to avoid those problems from the beginning. The functionality of the neobladder could be improved and the complication rate decreased. This is one of the major preexisting shortcomings after an orthotopic neobladder in the female. It seems to be even more important as the MS would most likely aggravate over time.

BSC mesh is a new, U-shaped small lightweight mesh for bilateral suspension. So far, during the more than 3-year follow-up, we did not demonstrate any major complications or POP. Urinary retention and mesh erosion did not occur. The latter could be a huge risk, in particular, for the neobladder and, thus, contradictory to mesh placement, in general. The BSC mesh seems to have optimal characteristics to avoid those problems from the beginning and is well suitable for laparoscopy also. The patient could empty the bladder as usual by abdominal squeezing combined with pelvic floor relaxation.

In this single case, the BSC mesh proved as a feasible option to maintain the functional integrity of the pelvic floor. Owing to the mesh properties and the simple fixation technique, the benefit–risk analysis seemed to be reasonable for this particular case. We could assure unproblematic neobladder function with good quality of life even in a patient with a severe neurologic comorbidity and potential subsequent limitations of pelvic floor functionality. We conclude that this could be part of a modular plan to protect female patients at risk from additional neocystocele problems.

Nevertheless, more experience, in particular, in females with underlying neurologic diseases and a longer follow-up also in non-neurologic patients are needed to prove the concept further.

## Conclusion

Laparoscopic cystectomy in female patients could be reasonably combined with laparoscopic sacrospinal fixation using a BSC mesh to maintain the functional integrity of both pelvic floor and neobladder.

## Abbreviations Used

BC: bladder cancer
BSC mesh: bilateral-sacrospinous-colposuspension mesh
CT: computed tomography
Cx: cystectomy
miBC: muscle invasive bladder cancer
MR: magnetic resonance imaging
MS: multiple sclerosis
OR: operating room
POP: pelvic organ prolapse
rCx: radical cystectomy
